# Cervical Electrical Neuromodulation Effectively Enhances Hand Motor Output in Healthy Subjects by Engaging a Use-Dependent Intervention

**DOI:** 10.3390/jcm10020195

**Published:** 2021-01-07

**Authors:** Hatice Kumru, África Flores, María Rodríguez-Cañón, Victor R. Edgerton, Loreto García, Jesús Benito-Penalva, Xavier Navarro, Yury Gerasimenko, Guillermo García-Alías, Joan Vidal

**Affiliations:** 1 Fundación Institut Guttmann, Institut Universitari de Neurorehabilitació Adscrit a la Universitat Autònoma de Barcelona, 08916 Badalona, Spain; vre@ucla.edu (V.R.E.); loretogarcia@guttmann.com (L.G.); jbenito@guttmann.com (J.B.-P.); xavier.navarro@uab.cat (X.N.); guillermo284@gmail.com (G.G.-A.); jvidal@guttmann.com (J.V.); 2 Universitat Autònoma de Barcelona, Bellaterra, 08193 Barcelona, Spain; 3 Fundació Institut d’Investigació en Ciències de la Salut Germans Trias i Pujol, 08916 Badalona, Spain; 4 Departament de Biologia Cel·lular, Fisiologia i Immunologia & Insititute of Neuroscience, Universitat Autònoma de Barcelona, and CIBERNED, Bellaterra, 08193 Barcelona, Spain; africa.flores@uab.cat (Á.F.); rodriguezcanonmaria@gmail.com (M.R.-C.); 5 Department of Integrative Biology and Physiology, University of California Los Angeles, Los Angeles, CA 90095, USA; 6 Pavlov Institute of Physiology, 199034 St. Petersburg, Russia; yuryg@ucla.edu; 7 Department of Physiology and Biophysics, University of Louisville, Louisville, KY 40292, USA

**Keywords:** transcutaneous spinal cord stimulation, hand training, combined intervention, neuromodulation, cervical spinal cord

## Abstract

Electrical enabling motor control (eEmc) through transcutaneous spinal cord stimulation is a non-invasive method that can modify the functional state of the sensory-motor system. We hypothesize that eEmc delivery, together with hand training, improves hand function in healthy subjects more than either intervention alone by inducing plastic changes at spinal and cortical levels. Ten voluntary participants were included in the following three interventions: (i) hand grip training, (ii) eEmc, and (iii) eEmc with hand training. Functional evaluation included the box and blocks test (BBT) and hand grip maximum voluntary contraction (MVC), spinal and cortical motor evoked potential (sMEP and cMEP), and resting motor thresholds (RMT), short interval intracortical inhibition (SICI), and F wave in the abductor pollicis brevis muscle. eEmc combined with hand training retained MVC and increased F wave amplitude and persistency, reduced cortical RMT and facilitated cMEP amplitude. In contrast, eEmc alone only increased F wave amplitude, whereas hand training alone reduced MVC and increased cortical RMT and SICI. In conclusion, eEmc combined with hand grip training enhanced hand motor output and induced plastic changes at spinal and cortical level in healthy subjects when compared to either intervention alone. These data suggest that electrical neuromodulation changes spinal and, perhaps, supraspinal networks to a more malleable state, while a concomitant use-dependent mechanism drives these networks to a higher functional state.

## 1. Introduction

Electrical stimulation of the spinal cord is an emerging valuable tool in clinical practice for facilitating the recovery of sensory and motor function in subjects with spinal cord injury (SCI) [1]. Outstanding clinical achievements have been recently reported showing that lumbar epidural stimulation facilitates the recovery of posture, stepping, and voluntary control of the lower limbs in subjects with chronic SCI [2,3,4,5,6]. Cervical epidural stimulation increased hand grip force and hand volitional control in tetraplegic patients [7]. On the other hand, recently developed non-invasive transcutaneous electrical spinal cord stimulation (electrical enabling motor control, eEmc) has demonstrated its efficacy to improve lower limb motor function after paralysis [8,9,10], as well as hand grip strength and voluntary control in tetraplegic patients [11,12,13,14]. In both stimulation conditions, the underlying hypothesis states that the spinal sensory–motor networks above, within, and below the lesion are neuromodulated and raised into an elevated functional state that enables and amplifies voluntary motor control [11,12,13,14]. In the context of SCI rehabilitation, the term eEmc [8,9,10] emphasizes and distinguishes this technique from other transcutaneous electrical stimulation approaches that directly induce muscular contraction, instead of facilitating and enabling voluntary control.

Since transcutaneous spinal cord stimulation (tSCS) is a non-invasive and safe method, it can be easily applied in healthy subjects and can also be employed to unravel the neurophysiological mechanisms acting on sensory and motor recovery in individuals suffering from SCI [12]. By applying high-frequency trains of stimuli (i.e., eEmc) to the thoracolumbar segments, locomotor-related neuronal networks can be neuromodulated to physiological states that enable the generation of bilateral rhythmic step-like movements with the legs placed in a gravity-neutral position in non-injured subjects [15] and in spinally injured subjects categorized as a complete SCI (ASIA A) [16]. One of the mechanisms considered to be important in that movement is mediated by activation of the spinal central pattern generators of locomotion [17]. The degree to which this specific mechanism contributes to upper limb movements, however, remains unclear, since it may act together with other adaptations to allow the recovery of a wide range of movements that are not predominantly repetitive, such as stepping. Importantly, continuous eEmc delivery also improves hand motor performance after one single exposure from 20 min to 2 h in SCI patients [11,12,13,14]. A recent work reveals that high-frequency eEmc induces plastic changes on neuronal circuits controlling upper limb function also in intact subjects [14].

Activity-dependent plasticity is the key phenomenon thought to underlie functional recovery observed after both physical activity and stimulation-based rehabilitative approaches in SCI [18,19,20]. However, the mechanisms recruited by physical training and by electrical stimulation, although partially overlapping, may involve different and perhaps synergistic processes leading to more effective neural circuit reorganization. However, reported studies do not address whether eEmc applied alone or together with hand training showed better clinical or neurophysiological effects in SCI patients. Indeed, the majority of the studies only applied eEmc combined with hand training [9,10,12,13]. More studies are needed to directly assess the role of use-dependent mechanisms in an attempt to optimize therapeutic efficacy of stimulation approaches and training. Here we hypothesized that the application of eEmc together with hand grip training can increase hand motor output more than either of the two interventions alone. We further hypothesized that the improved function would be the result of a synergistic reorganization of both spinal and supraspinal networks that could enable the performance of the neuromuscular unit being engaged. To answer these questions, each participant was subjected to a hand motor functional and neurophysiological assessment before and after each of three interventions (training, eEmc, and training + eEmc). To detect hand muscle strength and a manual dexterity, we used the hand grip maximum voluntary contraction (MVC) and the box and blocks test (BBT). Spinal cord excitability was assessed by F wave and by recruitment curves of spinal motor evoked potentials (sMEPs) in hand and arm muscles induced by single-pulse tSCS. Corticospinal excitability was measured by resting motor threshold (RMT) and cortical motor evoked potentials (cMEPs) in hand muscles induced by transcranial magnetic stimulation (TMS), and changes in intracortical inhibitory circuits were tested by short interval intracortical inhibition (SICI).

## 2. Experimental Section

### 2.1. Participants

**Figure 1:**
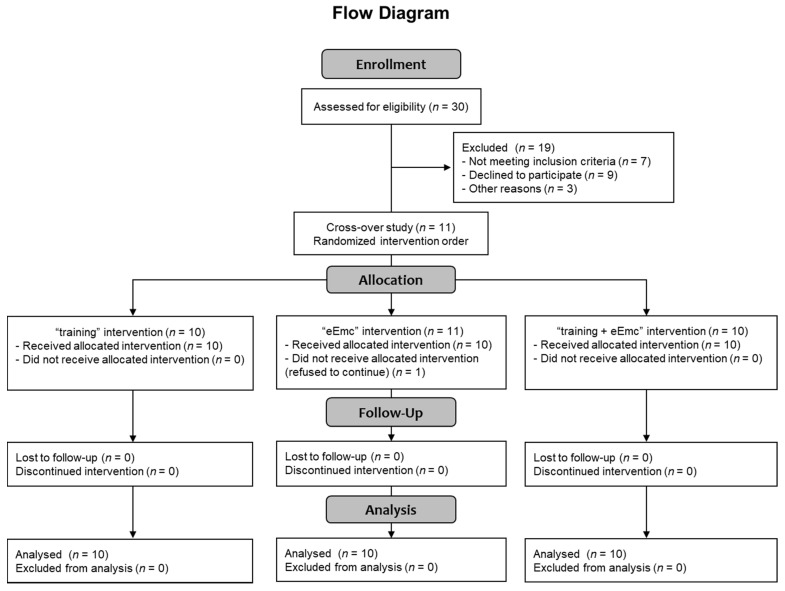
CONSORT 2010 flow diagram showing the number of subjects involved in each phase of the study.

Thirty healthy subjects were selected for participating in this study (Consolidated Standards of Reporting Trials (CONSORT) flow diagram on Figure 1). Recruitment of participants consisted of self-selection within the current users of our installations as well as asking the participants for referrals. Eleven healthy volunteers accepted informed consent and completed inclusion criteria with no known history of neurological disorders and accepted to participate in the study, and ten of them (7 men, 3 women; mean age = 38 ± 11 years, age range: 24–60 years; Table 1) completed all three interventions, and were included in the data analysis (CONSORT flow diagram on Figure 1). One subject rejected to continue at the beginning of the study because of unpleasant sensation through cervical electrical stimulation. Inclusion criteria were: male or female between 18 and 65 years old without any neurological disorder and other disorder which could limit the experiment (uncontrolled cancer, arthritis, etc.), and had written informed consent. Exclusion criteria were any metal implants, implanted electrical devices, medications that could raise seizure threshold, cardiac conditions, and history of syncope or concussion with loss of consciousness, tinnitus, or pregnancy. The study protocol was approved by the Research Ethics Committee of the Institute Guttmann and was conducted in accordance with the Declaration of Helsinki [21]. 

### 2.2. Experimental Design

It was a randomized crossover study, which included three different interventions. Interventions consisted of: (i) hand grip training, (ii) eEmc, or (iii) eEmc combined with hand grip training. All participants received each intervention once with at least 1 week between each intervention.

For hand functional outcomes, we assessed MVC during hand grip force and BBT. For neurophysiological assessment at spinal cord level, we evaluated F wave of abductor pollicis brevis (APB) muscle and recruitment curves of sMEPs in hand and arm muscles induced by single-pulse tSCS, applied to C3–C4 and then to C6–C7. At cortical level: RMT and cMEPs induced by TMS in hand muscles at 120% of RMT and SICI were recorded. All neurophysiological recordings were done in the dominant upper extremity muscles. The functional and neurophysiological assessments were realized at baseline, just after intervention (starting at 0′ post) and one hour after the end of intervention (starting at 60′ post, follow-up) to evaluate the possible short- and long-lasting aftereffects of each intervention. The duration of each experiment was around 4 h including the set up. Each complete functional and neurophysiological assessment took 30–45 min for each timepoint and was carried out in the following order: BBT, MVC, F wave, cortical RMT, cMEPs, SICI, cMEP recruitment curve, spinal RMT, and sMEP recruitment curve.

### 2.3. Functional Assessment

#### 2.3.1. Maximum Voluntary Contraction during Hand Grip Strength

The hydraulic hand grip dynamometer (Jamar Model 5030J1, Sammons Preston, NJ, USA) was used for MVC. Participants were asked to perform MVC for 4 s, as soon as they noticed a triggering stimulus, which was a mild electrical pulse at an intensity of 3 mA with 0.5 ms of duration delivered with ring electrodes to the fifth finger of the dominant hand. We recorded the maximal and maintained force during these 4 s in three consecutive trials, with at least one minute of rest between each trial. We registered the electromyographic (EMG) activity from the arm and hand muscles.

#### 2.3.2. Box and Blocks Test

This is a test of manual dexterity consisting of a box with a center partition [22]. The participant must pick up the maximum number of small cube-shaped blocks, one at a time, and drop them at the other side of the partition in 60 s. The score of BBT is represented by the number of blocks transported.

### 2.4. Neurophysiological Assessment

#### 2.4.1. Electromyographical Recording

EMG activity was recorded with a conventional EMG machine (Medelec Synergy, Oxford Instruments; Surrey, UK). After standard skin preparation, disposable adhesive surface electrodes (outer diameter of 20 mm; Technomed) were placed over the muscle belly of the APB, abductor digiti minimi (ADM), flexor carpi radialis (FCR), extensor digitorum (ED), and biceps brachii (BB) muscles of the dominant arm, with the cathode proximal and the anode approximately 2 cm distally. The EMG signal was amplified and then filtered using a band-pass of 30 Hz–10 kHz, amplitude sensitivity of 0.1–0.5 mV and epochs of 100 ms sweep duration and recorded at a sampling rate of 50 kHz. During assessments, we ensured that the baseline EMG activity of all recorded muscles was lower than 50 µV of amplitude before delivering each single stimulus: EMG activity was checked out online and, if necessary, the subject was reminded to be relaxed. If any background EMG activity was observed after stimulus delivery, this recording was eliminated in situ and stimulation procedure was repeated. EMG signals were stored in a Synergy computer and analyzed offline with MATLAB.

#### 2.4.2. F Wave

To measure the spinal motoneuron pool excitability, F wave was recorded in APB muscle using suprathreshold electrical median nerve stimulation at the wrist level [23,24]. Both stimulus delivery and EMG signal recording were processed by a conventional EMG machine (Medelec Synergy, Oxford Instruments; Surrey, UK). Electrical stimuli of 0.5 ms of duration were delivered at 1 Hz and the electrical intensity was set at supramaximal level to induce the maximal amplitude in M wave (Mmax) (range: 17–30 mA) [24]. A minimum of 10 stimuli were delivered to calculate F wave persistency [25].

#### 2.4.3. Recruitment Curve of sMEPs

To assess spinal cord excitability, we used monophasic rectangular 1-ms single-pulse tSCS delivered through 2 cm diameter hydrogel adhesive electrodes (axion GmbH, Hamburg, Germany) as cathodes at C3–C4 and then at C6–C7 and two 5 × 12 cm rectangular electrodes placed symmetrically over the iliac crests as anodes. The electric intensity for spinal RMT in APB muscle of the dominant hand was determined at baseline condition in each intervention. RMT was defined as the lowest intensity that elicited sMEPs of ≥50 μV peak-to-peak amplitude at APB muscle in at least 5 out of 10 consecutive trials. Recruitment curves of sMEPs were established at gradual increasing intensities from 90% to 150% of RMT (at 10% increments, three recordings at each intensity) in all recorded muscles of the dominant hand [14,26].

#### 2.4.4. Transcranial Magnetic Stimulation

Changes in corticospinal excitability and SICI were evaluated using a Magstim^®^ BiStim^2^ TMS (Magstim Company, Whitland, Wales, UK). Subjects were seated in a chair, resting their pronated forearms on a desk in front of them and were asked to stay relaxed but awake throughout the assessments. A figure-of-eight coil was held tangentially to the scalp over the motor area of the dominant hand in the optimal position for activating the APB in a posterior-anterior current direction. The hot point for evoking the largest cMEP in APB was marked over scalp. The following parameters were measured before, after, and during follow-up of each intervention: 1. RMT, defined as the lowest intensity of TMS that evoked a cMEP of ≥50 μV peak-to-peak amplitude in the APB muscle in at least 5 of 10 consecutive trials. 2. Mean amplitude of cMEPs using single-pulse TMS at 120% of RMT of ABP in five recordings. 3. SICI using paired-pulse TMS with a subthreshold conditioning stimulus (80% of RMT) and a suprathreshold test stimulus (120% of RMT) at interstimulus interval of 2 ms in 5 recordings without background activity (between 1 to 5 recordings for each subject were rejected because of background activity) [27]. 4. Recruitment curves were obtained at increasing intensities from 90% to 150% of RMT of APB, at 10% increments (three recordings at each intensity). The absence of baseline activation was verified before carrying out each of the abovementioned recordings.

### 2.5. Interventions

The study consisted of three interventions: (1) hand training, (2) eEmc, (3) eEmc combined with hand training. There was at least 1 week between each experiment.

**Figure 2:**
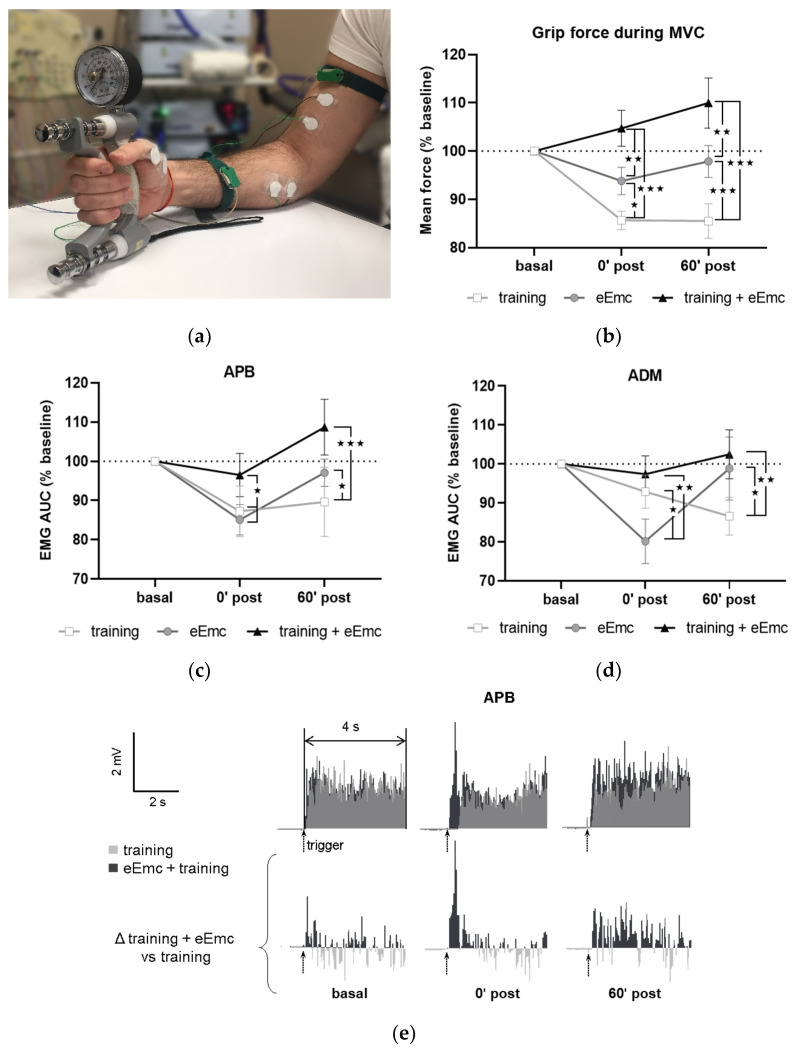
Maximal voluntary contraction (MVC) during hand grip strength. (**a**) Picture showing general setting and position for hand grip MVC assessment. (**b**) Percentage of MVC vs. baseline levels at 0 min (0′ post) and 60 min (60′ post) after each intervention. Significant differences between interventions: ★ *p* < 0.05, ★★ *p* < 0.01, ★★★ *p* < 0.001. (**c**,**d**) Percentage of area under the curve (AUC) of electromyographic (EMG) activity vs. baseline levels from APB (**c**) and from ADM (**d**). Significant differences between interventions: ★ *p* < 0.05, ★★ *p* < 0.01, ★★★ *p* < 0.001. (**e**) Representative APB muscle EMG registry (obtained from subject #5) corresponding to maximum voluntary contraction (MVC) trials during training and eEmc with training interventions at basal, 0′ and 60′ after intervention. Upper panels correspond to rectified values, and lower panels show difference between registers of both interventions for better difference appreciation. APB, abductor pollicis brevis; ADM, abductor digiti minimi.

Hand training protocol was adapted from previous studies [12] and consisted in grasping a hand grip dynamometer at maximum maintainable contraction for 20 s, followed by an 80-s resting period. This was repeated alternating right and left hands for 30 min (9 times per hand). Thus, the total duration of contractions for each treatment was 3 min for each hand and a total of 9 min of maximum effort for all three treatments for each muscle group. Mean sustained force was registered for each contraction. In all cases, the subjects were instructed and closely monitored to assure that a neutral wrist position and a 90° angle of the elbow was maintained while performing MVC with hand grip (Figure 2a).

A second condition consisted of delivering eEmc in the same time pattern (20 s of stimulation followed by an 80-s resting period for 30 min) in absence of hand training. eEmc was carried out with the transcutaneous electrical stimulator BioStim-5 (Cosyma Inc., Moscow, Russia). Previous reports indicate that applying stimulation simultaneously at two sites within the cervical area is consistently more effective than a single stimulation site [12]. Thus, we delivered eEmc simultaneously at two sites along the midline between spinous processes C3–C4 and C6–C7 during the corresponding intervention period. Regarding the possible mechanisms involved, it is critical to recognize that the intensity of stimulation at each spinal level was set at 90% of RMT induced by single-pulse tSCS at APB muscle of the dominant hand (range: ~30–80 mA, Table 1). Stimulation was continuously delivered using 2 cm diameter hydrogel adhesive electrodes (axion GmbH, Hamburg, Germany) as cathodes and two 5 × 12 cm rectangular electrodes placed symmetrically over the iliac crests as anodes. eEmc employed biphasic rectangular 1-ms pulses, each one filled with a carrier frequency of 10 kHz (i.e., each 1-ms pulse was composed of ten 0.1-ms biphasic rectangular pulses), that were delivered at a frequency of 30 Hz [13,17,28]. During stimulation, the subjects reported a non-painful but uncomfortable tingling sensation down the arms and at the site of stimulation, with some associated tonic contraction of paraspinal and posterior neck muscles.

The last condition was eEmc combined with hand training. The subjects performed maximum sustainable contractions and simultaneously received eEmc, alternating right and left hands for 30 min. The abovementioned tonic contraction of neck muscles during stimulation did not interfere with performance of repeated grip contractions, since force levels achieved in the dynamometer during this intervention were similar to those achieved during hand training without stimulation and participants reported no impediments to perform the training.

### 2.6. Data Analysis and Statistics

For each assessment, we calculated the mean ± standard error measurement (SEM) at baseline, right after and sixty minutes after intervention (0′ post and 60′ post intervention respectively) in each condition. We calculated the % changes according to baseline for each timepoint evaluation when appropriate. BBT, cMEP, SICI, and cMEP slopes could be easily compared between interventions without this normalization, so absolute values were preserved. The analysis of these outcome measures revealed the same effects if normalized.

For analyzing EMG activity during MVC, the area under the curve (AUC) in EMG of each muscle was calculated from the beginning of EMG signal during 4 s of MVC.

For the F wave analysis, we measured the peak-to-peak amplitude and for Mmax we measured the maximum amplitude of M wave. F wave was considered if peak-to-peak amplitude was at least 1% of M wave (Mmax) amplitude and F persistence was calculated by dividing the number of F responses by the number of stimuli from 10 recordings. The maximum amplitude of F was normalized to Mmax amplitude to obtain Fmax/Mmax ratio.

For recruitment curve of sMEPs and cMEPs, we measured the peak-to-peak MEP amplitude (μV) induced in each recording of all muscles and then we calculated the mean amplitude of MEPs from three recordings for each intensity for each subject and for each experimental condition. To detect the possible influence of each intervention and counteract the masking effect of interindividual variability, recruitment curves obtained at 0′ post and 60′ post intervention were normalized to baseline recruitment curves (which were considered as 0% change for all intensities) registered for each session and each subject. As a measure of corticospinal excitability, in addition, slope of non-normalized recruitment curves was calculated through linear regression from 90% to 150% RMT intensities, an interval that fitted linear distribution in all conditions and muscles [29].

For calculating SICI percentage, averaged peak-to-peak amplitude of the conditioned cMEP (obtained after the conditioning stimulus of 80% RMT with 2 ms separation from conditioned stimulus of 120% RMT) was expressed as a percentage of the averaged amplitude of the test cMEP (obtained at supramaximal 120% RMT stimulus): % = (conditioned cMEP/test cMEP) × 100.

Data were expressed as mean ± SEM. Shapiro–Wilk test was used to assess whether data were normally distributed or not. All sets of data fulfilled normal distribution requirement except F wave persistence, cortical RMT, SICI, and cMEP slope. For normally distributed data, repeated measures two-way analysis of variance (ANOVA) was used. For post-hoc analysis, we used Tukey’s test. For not normally distributed data, Friedman’s analysis was used followed by post-hoc Dunn’s test. Least squares regression was applied as linear regression method to calculate slope of sMEP and cMEP recruitment curves. Significance level was set at *p* < 0.05 in all cases.

## 3. Results

### 3.1. Hand Motor Output

Figure 2a shows a general setting for MVC assessment. MVC at baseline was 30.6 ± 0.9 kg. This value was consistent between sessions (within-subject variation coefficient was 10.9 ± 1.2%), although inter-subject variability was considerable (between-subjects variation coefficient was 28.7 ± 3.0%). Thus, all values were normalized to baseline for further analysis. Repeated-measures ANOVA showed that intervention (F_(2, 18)_ = 11.2, *p* < 0.001) and timepoint (F_(2, 18)_ = 3.67, *p* < 0.05) factors, as well as their interaction (F_(4, 36)_ = 8.77, *p* < 0.001) had a significant effect in MVC. MVC tended to increase following eEmc combined with training and did not change after eEmc alone, whereas training alone reduced MVC significantly after intervention during follow-up (*p* < 0.001 according to Tukey’s post hoc). The percentage of MVC vs. baseline levels was bigger after eEmc with training compared to training alone at 0′ and 60′ post intervention (*p* < 0.001 for each comparison) and also compared to eEmc intervention alone (*p* < 0.01 for each comparison) (Figure 2b).

The EMG activity of APB and ADM changed significantly according to two-way ANOVA (F_(1, 52)_ = 16.43, *p* < 0.001 and F_(1, 52)_ = 6.77, *p* < 0.01, respectively) (Figure 2c,d). There was a significant interaction between intervention and timepoint factors for APB and ADM muscles (F_(4, 36)_ = 3.36, *p* < 0.05 and F_(4, 36)_ = 4.34, *p* < 0.01, respectively). Tukey’s test showed that the training condition significantly reduced EMG activity of APB (at 0′ and 60′ post training) and ADM (at 60′ post intervention) (*p* < 0.05). Moreover, EMG activity in APB and ADM was significantly lower following training or eEmc alone than following eEmc with hand training at both 0′ and 60′ post-intervention (*p* < 0.05 at least for each comparison) (Figure 2c,d). The EMG of FCU, ED, or BB muscles did not change significantly by intervention or timepoint according to two-way repeated measures ANOVA. Figure 2e shows a representative EMG of APB muscle (subject #5) corresponding to MVC trials during “training” and “eEmc with training” interventions. This figure depicts similar EMG activity levels at basal conditions before these two interventions, but higher EMG activity at both 0′ post and 60′ after the combined intervention, an effect that becomes obvious when calculating the difference in EMG activity between “eEmc with training” and “training” condition.

**Figure 3:**
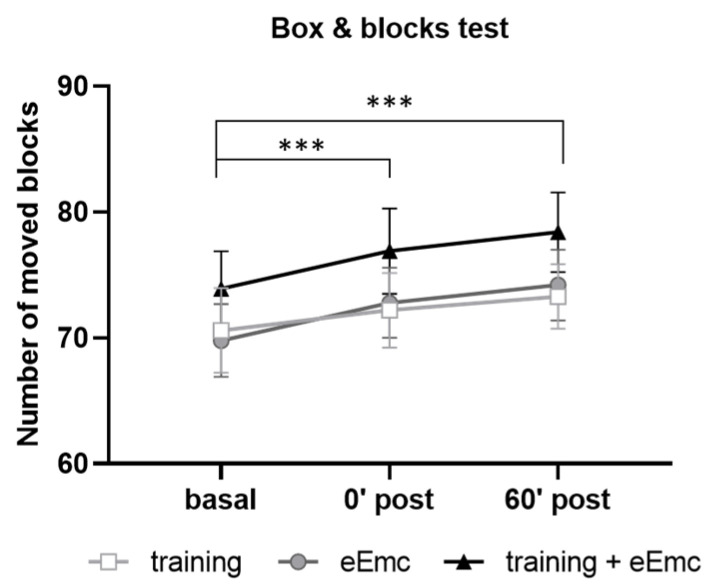
Number of moved blocks in the box and blocks test (BBT) for hand training, eEmc, or combination of both interventions right after intervention (0′ post) and during follow-up (60′ post) in comparison to basal evaluation. Significant differences between timepoints according to Tukey’s multiple comparisons (considering all intervention groups as a whole): *** *p* < 0.001.

Manual dexterity was assessed with the BBT in order to detect possible alterations induced by power grip training and/or eEmc-based interventions. Two-way ANOVA did not show an effect of intervention (F_(2, 18)_ = 2.62, *p* = 0.10) neither timepoint × intervention interaction (F_(4, 36)_ = 0.206, *p* = 0.93). However, there was a significant timepoint effect (F_(2, 18)_ = 43.5, *p* < 0.001) with increasing number of boxes, being significantly higher just after intervention (0′) and during follow-up (60′) in comparison to basal evaluation when equally considering all interventions (*p* < 0.001) (Figure 3).

### 3.2. Spinal Cord Excitability

**Figure 4:**
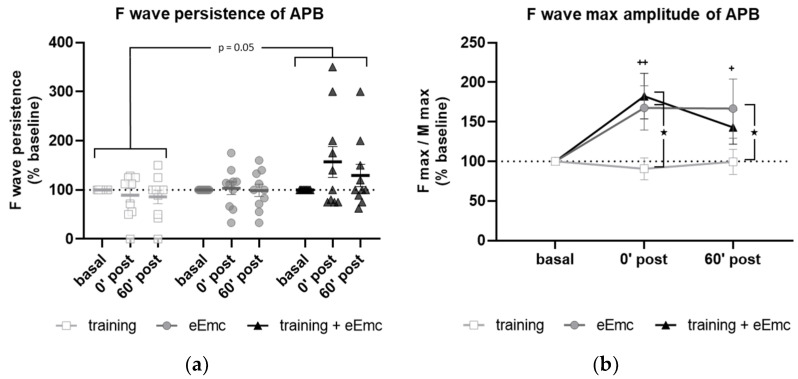

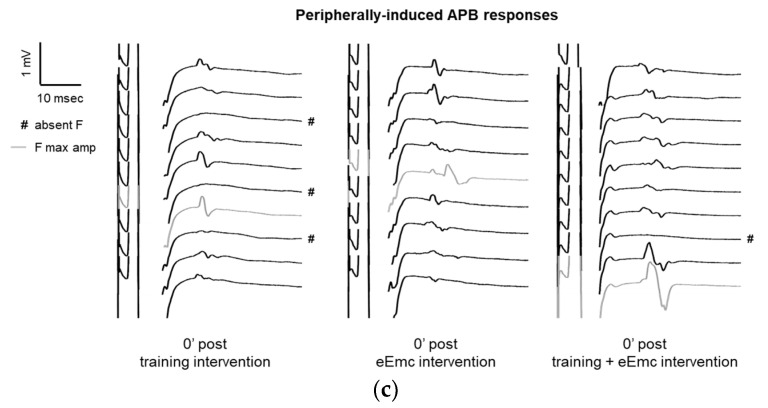
Variables of F waves in APB by median nerve stimulation. (**a**) F wave persistency. Significant difference between eEmc with training and training alone (regardless of the timepoint): *p* = 0.05. Data expressed as median ± interquartile range. (**b**) Averaged maximum amplitude of F wave for each condition and timepoint. Significant differences vs. baseline: ^+^ *p* < 0.05 (for training + eEmc), ^++^ *p* < 0.01 (for eEmc alone), and between interventions: ★ *p* < 0.05. (**c**) Representative APB muscle registers (obtained from subject #7) corresponding to ten consecutive median nerve stimulations showing M and F wave at 0′ after in each intervention. Light grey registers highlight F waves with maximum amplitude. # Symbol indicates registers where no F wave presence was considered. APB, abductor pollicis brevis.

F wave was used to detect excitability of anterior horn motoneurons in the spinal cord. There was a significant effect of intervention over F wave persistence (Friedman’s test = 5.84, df 3.30, *p* = 0.05) (Figure 4a). Dunn’s post-hoc comparisons revealed an overall increase in F persistence after eEmc combined with training compared to training alone that almost reached statistical significance (*p* = 0.053). The intervention factor had a nearly significant impact on F wave amplitude (F_(2, 18)_ = 3.10, *p* = 0.07). Post hoc comparisons showed that eEmc delivery, with or without hand training, increased F wave amplitude when compared to training alone (*p* < 0.05), particularly at 0′ post intervention (Figure 4b,c).

Excitability of spinal networks was tested by single-pulse tSCS at cervical level. The RMT in APB at baseline was of 51.0 ± 2.9 mA at C3–C4 and 60.8 ± 3.5 mA at C6–C7. The recruitment curve of sMEP from upper extremity muscles did not show any significant changes in any muscle at any intensity and in any intervention (*p* > 0.05) (Table S1).

### 3.3. Corticospinal Excitability and Intracortical Inhibition

**Figure 5:**
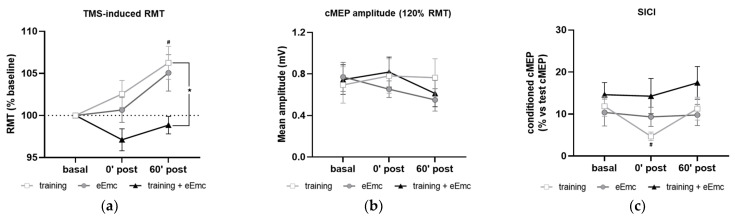
Effect of hand training, eEmc and eEmc + training on the corticospinal excitability and SICI. (**a**) Percentage of resting motor threshold (RMT) vs. baseline levels in APB at 0 min (0′ post) and 60′ min (60′ post) after intervention. Significant differences between interventions: ★ *p* < 0.05, (training vs. eEmc + training), and in comparison to baseline: # *p* < 0.05 (for training). (**b**) Effect of training, eEmc and eEmc + training on the supramaximal cMEP amplitude (120% transcranial magnetic stimulation (TMS)) according to RMT. (**c**) SICI: (average of conditioned cMEP) × 100/ (average of test cMEP) recorded at APB muscle and obtained at 0′ and 60′ after each intervention. # *p* < 0.05 for training at 0′ post in comparison to baseline. APB, abductor pollicis brevis; RMT, resting motor threshold; cMEP, cortically-induced motor evoked potential; SICI, short-interval intracortical inhibition.

The RMT in APB at basal conditions was 37.9 ± 1.2% of maximum TMS intensity. RMT was significantly affected by timepoint in the training intervention (Friedman’s test = 7.93, df 3.10, *p* < 0.05) and by intervention at 60′ post (Friedman’s test = 8.06, df 3.10, *p* < 0.05) (Figure 5a). Dunn’s post-hoc comparisons confirmed that higher RMT was found 60′ after training (*p* < 0.05). eEmc with training significantly reduced RMT when compared to training alone at this timepoint (*p* < 0.05). Corticospinal excitability measured by cMEP amplitude obtained at 120% RMT stimulus did not differ between interventions (F_(2, 18)_ = 0.25, *p* = 0.77) or between timepoints (F_(2, 18)_ = 1.55, *p* = 0.23) (Figure 5b).

SICI was significantly affected by timepoint in the training intervention (Friedman’s test = 7.40, df 3.10, *p* < 0.05). Dunn’s post hoc analysis revealed that hand training induced higher inhibition (i.e., smaller conditioned cMEP amplitude) at 0′ post-intervention (*p* < 0.05) compared to baseline, with no other significant comparisons (Figure 5c).

**Figure 6:**
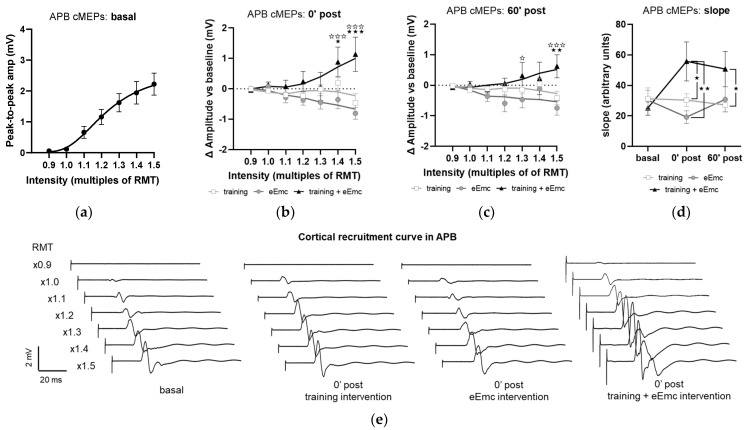
Effect of interventions on cortical recruitment curve. (**a**) Pooled APB recruitment curves obtained at baseline for each subject and intervention. (**b**,**c**) Increase or decrease (in millivolt (mV); indicated as Δ) in APB recruitment curves with respect to baseline at 0′ (**b**) and 60′ (**c**) post intervention. ★ *p* < 0.05, ★★ *p* < 0.01, ★★★ *p* < 0.001 training vs. training + eEmc; ✰ *p* < 0.05, ✰✰✰ *p* < 0.001 eEmc vs. training + eEmc. (**d**) Slope of APB cMEP recruitment curves. Indicated slopes correspond to the slope of the linear regression line through stimulation intensities (in %RMT) and cMEP values (in mV), multiplied ×1000 to facilitate data visualization. ★ *p* < 0.05, ★★ *p* < 0.01, between interventions at 0′ post and 60′ post. (**e**) Representative APB muscle recording (obtained from subject #1) corresponding to recruitment curve assessment at basal (three conditions averaged) and 0′ post each intervention. APB, abductor pollicis brevis; RMT, resting motor threshold; cMEPs, cortically-induced motor evoked potentials.

The recruitment curves of cMEP at baseline did not significantly differ between interventions in any muscle and appear represented as a single pool in Figure 6a, which shows APB data. Analysis of APB muscle recruitment curves normalized to baseline showed significant intervention × timepoint interaction after intervention (F_(12, 108)_ = 3.59, *p* < 0.001) and during follow-up (F_(12, 108)_ = 1.58, *p* < 0.05). Specifically, eEmc with training enhanced cMEP amplitude significantly in comparison to training and eEmc alone at high TMS-stimulation intensities (1.4× and 1.5× RMT at 0′ post-intervention and 1.3× and 1.5× RMT at 60′ post-intervention; Figure 6b,c). Corticospinal excitability was also quantified by calculating the slope of non-normalized absolute recruitment curves, which also resulted in a significant effect of intervention (Friedman’s test = 8.08, df 3.30, *p* < 0.05), with higher slopes for APB after eEmc with training compared to training or eEmc alone at 0′ and 60′ post-intervention, according to Dunn’s multiple comparisons (Figure 6d, Table S2). There were no significant differences for slopes analyzed for other muscles (Table S2).

## 4. Discussion

The present study shows that one single session of cervical eEmc modifies the excitability of neuronal networks controlling upper limb function in healthy subjects. These changes strongly depend on eEmc combined with hand training, improving hand grip force and increasing spinal and corticospinal excitability in comparison to each intervention tested alone. Indeed, eEmc alone increased cervical spinal cord excitability measured by anterior horn motoneuron excitability, but had no effect on corticospinal excitability or hand grip force, while hand training alone reduced hand grip force, corticospinal excitability, and intracortical inhibition. The effects of eEmc combined with hand training appeared immediately after the intervention and were observed in hand grip force measured by MVC, in spinal cord excitability measured by F wave, and in corticospinal excitability measured by RMT induced by TMS and cMEP recruitment curve, and these effects lasted at least one hour in most clinical and neurophysiological assessments.

Previous studies in SCI patients have reported improved hand motor performance following eEmc, even after one single session [11,12,13,14,17]. However, it is not clear whether eEmc benefits or not from simultaneous physical training to deploy its full potential to facilitate motor improvement. Indeed, although eEmc has been mostly delivered together with training, improvements were reported in hand function immediately after eEmc in absence of any hand training and persisted for at least 75 min following 20 min of 30 Hz stimulation [14].

Diverse modalities of spinal cord stimulation, traditionally employed to treat chronic pain, interfere with proprioceptive and sensitive processing [30]. The lack of differences observed between interventions in the BBT indicates that eEmc does not interfere with manual dexterity as might be observed if stimulation altered sensorial/proprioceptive processing or precise motor control. Of note, progressive improvement shown after repeated BBT testing in all groups suggests some form of motor or cognitive learning that allows improved task performance, and importantly, this process is not compromised by eEmc exposure either. As expected, however, we did not observe any improvement after eEmc exposure since little space for dexterity refinement is left in uninjured participants at this particular task.

Unlike eEmc or the combined treatment, hand training alone resulted in a significant decrease in hand grip force as shown by reduced MVC, an effect that lasted at least one hour. The decline in hand grip force was partially correlated with changes in EMG activity of the hand muscles APB and ADM but not with more proximal arm muscles (Figure 3b,c). This was probably due to fatigue at peripheral and/or central level, since the voluntary effort performed by all subjects was maximal although it did not reach the force level achieved in basal conditions, accordingly to previous observations in similar settings (reviewed in [31]). This was unsurprising due to the nature of the intervention (i.e., repetitive maximal contractions for 30 min). Interestingly, whereas MVC strength remained relatively unaltered after eEmc delivery alone as no exercise-induced fatigue could be present, an increase in grip force was observed after eEmc together with training when compared to both hand training and eEmc alone, even 1 h after the intervention. This suggests that eEmc delivery, together with hand training, is probably counteracting the fatigue effect induced by concomitant exercise, but other mechanisms are required to explain the differences compared to eEmc alone. Moreover, the maintenance of EMG activity levels after the combined intervention suggests that eEmc requires concomitant hand training to more successfully sustain the recruitment of muscle fibers and muscle contraction levels. The fatiguing and the compensatory effects are likely to be a combination of neural and muscular elements [32].

F wave has been considered an indirect measure of motoneuron pool excitability [33] and reflects backfiring of a small number of motoneurons which are reactivated by antidromic impulses following supramaximal stimulation of a peripheral nerve. Increased Fmax/Mmax amplitude ratios (by means of increased Fmax and constant Mmax) observed after eEmc alone, with or without hand training, indicated that a higher portion of the motoneuron pool can be activated by an antidromic stimulus after eEmc intervention. We also observed higher F persistence when eEmc was applied simultaneously to hand training during the intervention. Whereas F max amplitude may reflect de maximal number of motoneurons backfiring simultaneously, F persistence may reflect the probability of these motoneurons to backfire at the same time. Since the probability to successfully produce an orthodromic action potential after antidromic depolarization depends on the refractory period of the axonic cone [34], it is possible that eEmc alone is sufficient to increase motoneuron excitability but somehow does not favor optimal refractory period conditions to increase F wave occurrence. Moreover, it has been shown that pre-activation increases the occurrence of F wave in neurons with a low number of F waves at rest, but not in those neurons with high basal number of F responses [35]. Considering this, a possibility might be that hand grip training focuses eEmc-induced excitability to a discrete spinal neuronal population, perhaps the one contributing to grip performance.

Previous reports show that tSCS at the cervical level generally reaches anterior horn motoneurons through activation of dorsal root afferents, as shown by elicited bilateral monosynaptic reflexes [36,37], although tSCS can however preferentially activate sensory or motor roots depending on the stimulation parameters and electrode location [38,39]. A recent work shows that eEmc applied at the cervical spinal cord is able to increase the excitability of spinal networks in uninjured and SCI participants [14], which goes in line with increased motoneuron excitability observed in our study. In the work of Benavides et al., sMEPs were elicited by cervicomedullary magnetic stimulation and showed increased amplitude in proximal and distal arm muscles for 75 min following tSCS, but not sham-tSCS. In contrast, we could not observe any difference between interventions on sMEPs elicited by single-pulse tSCS. A reason for this discrepancy could rely on the different current distribution within the spinal cord elicited by magnetic and electrical stimulation, or perhaps our single-pulse tSCS-based method to recruit upper limb muscles resulted not sensitive enough to detect such changes at the level of spinal network excitability. However, since eEmc delivers electrical currents at intensities below the RMT, an indirect (more than a direct) effect on cervical spinal networks may have increased motoneuron excitability.

Corticospinal excitability is known to be altered due to physical training and also to diverse modalities of CNS stimulation [14,24,31,40]. Both voluntary muscular activity (e.g., sustained maximal contractions) and “imposed” muscular activity (induced by sustained electrical stimulation at motor point) reaching fatiguing levels result in transiently decreased corticospinal excitability [40]. In agreement, we found increased TMS-evoked RMT after hand grip training and after eEmc without training, an effect that persisted at least 1 h after each intervention. TMS-evoked RMT, however, remained unaltered after eEmc with hand training. In addition, recruitment curves at APB muscle showed higher cMEP amplitude after eEmc with hand training than each intervention separately. Recruitment curves of cMEP from muscles other than APB did not show this enhancement, possibly because TMS was optimized to recruit APB. Increased cMEP amplitude in the APB recruitment curve entails that the number of spinal motoneurons recruited at particular TMS stimulation intensities is higher after eEmc with training than after each intervention alone. This could reflect changes at any level within the corticospinal motor pathway controlling the APB muscle: intracortical circuits favoring cortical primary motoneuron to depolarize, excitability of cortical motoneurons itself, axonal corticospinal conductivity, spinal premotor networks favoring spinal motoneuron depolarization, excitability of spinal motoneurons itself, peripheral axonal conductivity, or efficiency at the neuromuscular junction level [41]. Thus, increased spinal excitability itself reflected as F wave facilitation after eEmc, with or without hand training, could contribute to increased corticospinal excitability. This finding might also be explained by the different mechanisms underlying cMEP depression after exercise or after electrical stimulation. As discussed by Pitcher et al., cMEP depression may emerge as an adaptation to tonic afferent input to the cortex from the exercising muscle (presumably Golgi tendon organs or group III and non-spindle group II afferents [42]), altering cortical excitability without altering spinal excitability. On the other hand, peripheral electrical stimulation initially induces cMEP facilitation [43] until prolonged stimulation leads to cMEP depression reflecting cortical plasticity [40]. In our study, since electrical stimulation is applied at spinal level, and enhances spinal excitability, several concurrent (but opposing) effects might be taking place, and corticospinal output may depend on their final balance. Thus, a possible explanation for increased corticospinal output after eEmc with training includes that spinal cord stimulation could interfere with afferent inputs to the cortex from the exercising muscle, counteracting the effect of muscular activity.

Given that intracortical networks influence corticospinal output, and that exercise-induced decrease in corticospinal excitability has been ascribed to intracortical rather than spinal mechanisms [40], we assessed SICI to reveal the possible modulation of inhibitory circuits in the primary motor cortex [44]. We observed increased intracortical inhibition right after power grip training, which may be surprising given that fatiguing exercise usually leads to reduced inhibition [45,46]. However, this event has been reported *during* maximal contractions, being already absent at 7–10 min after ceasing the exercise. In addition, increased excitability of intracortical inhibitory circuits (i.e., increased SICI) occurs *after* relaxation from voluntary contractions [47], a phenomenon that has been interpreted as a rebound effect after relaxation. This may explain our observations and further supports our vision supporting that eEmc combined with training prevents its fatigue-induced effects. On the other hand, we could not find any effect of eEmc, with or without hand training, on intracortical inhibition. These results differ from a recent study reporting increased SICI after eEmc alone that lasted for more than 1 h [14]. Methodological differences might explain these divergences, such as duration of stimulation, frequency stimulation, and simultaneous evaluation of other parameters together with SICI in the present study. Notably, eEmc delivered simultaneously to hand training prevented the alterations observed in SICI after hand training alone. This might suggest, again, the possible prevention of fatigue induced by simultaneous eEmc, indicating that the modulation of intracortical inhibitory motor circuits by eEmc delivered with hand training contributes to increased corticospinal excitability observed after this intervention. However, further studies are needed to confirm this hypothesis by accurately controlling the time-course of SICI during and after each intervention.

Mechanisms of plasticity observed in unimpaired subjects will, to some extent, be extrapolable to individuals with complete and incomplete spinal cord injury (SCI), since a portion of neural substrates remains preserved after SCI. Indeed, even in very severe injuries with completely abolished motor and sensory function, some spinal axons survive at the epicenter of the injury [48,49]. Thus, SCI population may benefit not only from plastic changes taking place in spinal neurons but also from the possible enhancement of the descending motor drive through residual corticospinal projections (or other descending systems), and via the interneurons that form the propriospinal system forming intersegmental connections ipsilaterally and bilaterally. However, this benefit might be limited, particularly below the injury, by the amount of spared brain-to-spine connections.

Activity-based neural plasticity mechanisms involve both physiological (functional modification of existing synapses and neurones) and structural changes that alter the anatomical connectivity of neurones (circuit reorganization by means of formation, removal, and morphological remodeling of synapses, dendritic spines, and even neurites) (reviewed in [50]). In the present study, signs of neural plasticity were observed after a single training and/or stimulation session, suggesting that short-term plastic changes were taking place. These may primarily affect intrinsic properties of neurons to depolarize and generate an action potential, including, for instance, modulation or relocation of ion channels and surface receptors. Sustained and/or repeated exposure to this activity-dependent plasticity (based either on physical training, electrical stimulation, or both) may lead these short-term changes to promote occurrence of long-term plasticity involving mechanisms, such as long-term potentiation and depression, morphological changes of dendrites, synaptogenesis, and axonal branching/regeneration [20]. Based on our findings, rehabilitative strategies involving the simultaneous application of physical training and spinal electrical neuromodulation may have more chances to induce long-term plastic adaptations than either intervention alone.

### Limitations

We acknowledge diverse limitations present in the current study: (i) sample size was relatively small, which might compromise statistical power and subsequent interpretation of the results. Indeed, only a third of all screened candidates finally participated in the study (see Figure 1), and this was in great part due to candidate refusal to participate, probably because of concerns about discomfort associated to cervical stimulation, possible side effects of the intervention and limited availability of participants (each testing session required around 4 h to be completed). (ii) The duration of the testing sessions also resulted exhausting for participants, limiting the number of replicates that could be recorded for some neurophysiological parameters (recruitment curve, cMEP, SICI). This might increase variability in the observed data as well. (iii) Since many parameters were measured, it is possible that testing itself influenced those measured in the last place. Moreover, care should be taken when interpreting temporal patterns observed at 0′ and 60′ post-intervention, which may correspond to longer timepoints in fact since complete functional and neurophysiological assessment lasted 30–45 min. (iv) We did not assess afferent processing. For this reason, we cannot conclude about the possible roles of altered afferent pathways on the observed results.

## 5. Conclusions

As a summary, eEmc combined with hand training enhanced hand motor output in healthy subjects as observed in power grip performance when compared to hand training or eEmc applied separately. This effect was associated to increased corticospinal excitability that was due, at least in part, by plastic changes induced at spinal level and possibly at cortical level. These changes may also possibly reflect prevention of fatigue-associated alterations induced by physical training. Therefore, eEmc combined with use-dependent interventions should be considered when designing rehabilitation protocols for restoring motor performance following stroke, SCI, or other neurological affections compromising motor function.

## Figures and Tables

**Table 1:** Demographic data from the ten subjects participating in the present study. Stimulation intensities employed during high-frequency electrical enabling motor control (eEmc) are also shown.

Subject	Sex	Age	Hand Preference	eEmc Intensity (mA)	
				C3–C4	C6–C7
1	male	44	right	29	32–38
2	male	60	right	59–67	69–77
3	female	25	right	28–30	30–32
4	female	27	left	34	36
5	female	33	right	52–54	59–63
6	male	41	right	34–38	45
7	male	24	right	36–50	56–59
8	male	51	right	52–63	63–77
9	male	39	right	59	72–74
10	male	38	both	54–67	63–77

## Data Availability

The data presented in this study are available on request from the corresponding author. The data are not publicly available due to privacy restrictions.

## Supplementary Materials

The following are available online at https://www.mdpi.com/2077-0383/10/2/195/s1, Table S1: Slopes of recruitment curves of tSCS-elicited spinal MEPs for each analysed muscle, intervention and timepoint, Table S2: Slopes of recruitment curves of TMS-elicited cortical MEPs for each analysed muscle, intervention and timepoint.
