# Supplementary material for: Cervical Electrical Neuromodulation Effectively Enhances Hand Motor Output in Healthy Subjects by Engaging a Use-Dependent Intervention
Source: J Clin Med. 2021 Jan 7;10(2):195. doi: 10.3390/jcm10020195 (PMC7827883; doi:10.3390/jcm10020195)
Supplement: Supplementary file 1 [file jcm-10-00195-s001.pdf]

Journal of
Clinical Medicine

Supplementary material

Table S1

Slopes of recruitment curves of tSCS-elicited spinal MEPs for each analysed muscle, intervention and timepoint. Indicated slopes correspond to the slope of the linear regression line through stimulation intensities (in %RMT) and sMEP values (in mV), multiplied *1000 to facilitate data visualization. Data correspond to mean ± SEM. MEP, motor evoked potential; APB, abductor pollicis brevis; ADM, abductor digiti minimi; FCU, flexor carpi ulnaris; ECR, extensor carpi radialis; BB, biceps brachii.

**Slope of recruitment curves**		**Spinal C3-C4 MEPs**			**Spinal C6-C7 MEPs**		
		**training**	**eEmc**	**training + eEmc**	**training**	**eEmc**	**training + eEmc**
APB	basal	3,7 ± 1,4	5,4 ± 2,7	4,1 ± 1,6	3,9 ± 1,5	5,6 ± 2,4	4,0 ± 1,6
	0' post	3,7 ± 1,2	4,5 ± 2,7	4,3 ± 1,6	4,0 ± 1,4	4,5 ± 2,4	3,7 ± 1,4
	60' post	4,4 ± 1,6	4,4 ± 2,1	4,6 ± 1,4	4,0 ± 1,4	5,3 ± 2,3	3,8 ± 1,6
ADM	basal	5,1 ± 1,7	4,9 ± 1,8	4,2 ± 1,7	5,0 ± 1,8	5,6 ± 2,0	5,3 ± 1,9
	0' post	4,6 ± 1,6	4,3 ± 1,6	4,6 ± 1,6	5,6 ± 2,3	4,6 ± 1,9	4,5 ± 2,1
	60' post	5,0 ± 2,0	5,4 ± 2,0	3,9 ± 1,6	5,1 ± 2,2	5,9 ± 2,0	4,4 ± 2,1
FCU	basal	1,5 ± 0,3	1,7 ± 0,4	2,4 ± 0,6	1,7 ± 0,3	2,4 ± 0,5	2,5 ± 0,6
	0' post	1,9 ± 0,3	2,1 ± 0,6	2,0 ± 0,6	2,1 ± 0,3	1,6 ± 0,3	2,0 ± 0,4
	60' post	1,9 ± 0,4	1,8 ± 0,4	2,6 ± 0,5	1,6 ± 0,4	2,5 ± 0,6	3,0 ± 0,7
ECR	basal	2,9 ± 0,7	1,9 ± 0,3	2,2 ± 0,4	2,3 ± 0,6	2,7 ± 0,5	2,0 ± 0,5
	0' post	3,1 ± 0,8	2,3 ± 0,3	2,2 ± 0,4	2,9 ± 0,9	2,5 ± 0,8	2,1 ± 0,4
	60' post	2,9 ± 0,7	1,5 ± 0,2	2,2 ± 0,5	2,5 ± 0,5	2,4 ± 0,7	2,4 ± 0,7
BB	basal	1,2 ± 0,3	1,0 ± 0,2	1,9 ± 0,6	0,9 ± 0,2	1,0 ± 0,2	1,2 ± 0,3
	0' post	1,2 ± 0,3	1,0 ± 0,2	3,0 ± 1,6	1,0 ± 0,3	0,8 ± 0,2	1,3 ± 0,4
	60' post	1,0 ± 0,2	1,1 ± 0,2	1,7 ± 0,5	0,9 ± 0,2	0,9 ± 0,2	1,2 ± 0,3

Table S2

Slopes of recruitment curves of TMS-elicited cortical MEPs for each analysed muscle, intervention and timepoint. Indicated slopes correspond to the slope of the linear regression line through stimulation intensities (in %RMT) and cMEP values (in mV), multiplied *1000 to facilitate data visualization. Data correspond to mean ± SEM. *p<0.05 training vs eEmc with training, ††p<0.01 eEmc vs eEmc with training. MEP, motor evoked potential; APB, abductor pollicis brevis; ADM, abductor digiti minimi; FCU, flexor carpi ulnaris; ECR, extensor carpi radialis; BB, biceps brachii.

Slope of recruitment curves		Cortical MEPs			
		training	eEmc	training + eEmc	
APB	basal	31,3 ± 7,1	30,0 ± 6,7	25,2 ± 4,8	
	0' post	30,5 ± 4,2	19,4 ± 4,2	55,8 ± 12,8	*††
	60' post	27,3 ± 4,3	30,7 ± 8,2	51,0 ± 11,6	*
ADM	basal	15,9 ± 3,4	20,6 ± 3,9	19,9 ± 4,1	
	0' post	22,8 ± 4,5	20,4 ± 4,3	20,1 ± 3,5	
	60' post	20,8 ± 4,1	19,0 ± 4,8	17,2 ± 2,4	
FCU	basal	10,5 ± 3,6	8,1 ± 2,4	10,5 ± 3,6	
	0' post	6,5 ± 1,6	10,5 ± 2,7	9,7 ± 3,6	
	60' post	5,3 ± 1,0	8,9 ± 2,2	9,6 ± 2,8	
ECR	basal	10,9 ± 3,0	10,1 ± 2,2	9,1 ± 2,0	
	0' post	9,8 ± 1,6	10,6 ± 2,1	8,9 ± 2,0	
	60' post	11,1 ± 2,4	11,3 ± 3,8	12,4 ± 2,4	
BB	basal	1,8 ± 0,5	1,6 ± 0,6	3,6 ± 0,9	
	0' post	5,3 ± 1,4	2,5 ± 0,6	2,8 ± 1,8	
	60' post	4,0 ± 1,1	3,3 ± 1,4	4,3 ± 2,3	
